# Evaluation of Methanol-Dichloromethane Extract of *Stemonocoleus micranthus* Harms (Fabaceae) Stem Bark for Anti-Inflammatory and Immunomodulatory Activities

**DOI:** 10.1155/2020/1738163

**Published:** 2020-05-06

**Authors:** Florence Nwakaego Mbaoji, Akachukwu Marytheresa Onwuka, Sunday Onu, Ikechukwu Emmanuel Peter, Justus Amuche Nweze, Lilian Eleje Okonta

**Affiliations:** ^1^Department of Pharmacology & Toxicology, Faculty of Pharmaceutical Sciences, University of Nigeria, Nsukka 410001, Nigeria; ^2^Department of Science Laboratory Technology, Faculty of Physical Sciences, University of Nigeria, Nsukka 410001, Nigeria; ^3^Department of Pharmacognosy and Environmental Medicines, Faculty of Pharmaceutical Sciences, University of Nigeria, Nsukka 410001, Nigeria

## Abstract

**Background:**

The stem bark decoction of *Stemonocoleus micranthus* Harms (Fabaceae) is most widely used traditionally as a remedy for various diseases such as malaria and boil. In this study, the anti-inflammatory and immunomodulatory activities of the methanol-dichloromethane extract (MDE) from the stem bark of the plant in rodents were evaluated.

**Methods:**

The carrageenan-induced rat paw oedema, cotton pellet-induced granuloma in rat, and xylene-induced ear oedema in mice were used to study the anti-inflammatory activity of methanol-dichloromethane extract of *Stemonocoleus micranthus* (MDESm) (100, 200, and 400 mg/kg). The effects of MDESm (100, 200, and 400 mg/kg) on cyclophosphamide-induced immunosuppression, neutrophil adhesion, carbon clearance, and haematological and biochemical parameters were carried out to study its immunomodulatory activity in mice.

**Result:**

MDESm (100 mg/kg, p.o.) significantly (*p* < 0.05) inhibited carrageenan-induced oedema by 57.1% at 5^th^ h posttreatment compared with control. At 100 mg/kg, p.o., MDESm significantly (*p* < 0.05) reduced cotton pellet-induced granuloma by 39.28% and nonsignificantly reduced xylene-induced ear oedema by 34.1%. Treatment with MDESm (100 and 400 mg/kg) nonsignificantly abolished the neutropenia caused by cyclophosphamide with a percentage neutrophil reduction of 0 and −14.86%, respectively, while MDESm (200 mg/kg) and levamisole (50 mg/kg) had a nonsignificant reduction in neutrophil count (10.16 and 31.40%), respectively, all compared to the distilled water-treated group with a neutrophil count of −9.82%. MDESm at doses of 100 and 200 mg/kg increased phagocytic index by 0.0447 ± 0.00762 and 0.0466 ± 0.00703, respectively, although not significantly when compared to the control group with a value of 0.0226 ± 0.02117. There was a decrease in WBC and lymphocyte counts in MDESm- (200 mg/kg) treated group, suggesting immunosuppressive potential at this dose. MDESm caused a dose-dependent decrease in ALT and core liver enzymes, suggesting a hepatoprotective effect. The acute toxicity test revealed that MDESm is safe in mice with an oral lethal dose (LD_50_) of >5 g/kg.

**Conclusion:**

The methanol-dichloromethane extract of *Stemonocoleus micranthus* Harms possesses mild anti-inflammatory and immunomodulatory activities which may be more pronounced upon fractionation and purification. Therefore, more investigations are needed to explore these activities further.

## 1. Introduction

Inflammation is a broad term, but essentially it is the host defense reactions to tissue injury or infection caused by numerous stimuli such as chemicals, physical trauma as well as infectious agents. It is triggered by the immune system cells and characterized by dysfunction of the tissues and organs, leading to swelling, heat, redness, and pain [1]. The variations in the levels of immune system cells involved in inflammation have been reported to have consequential implications in the pathology and physiology (function and symptoms) of various diseases including diabetes, atherosclerosis, obesity, cancer, and fibrosis [2, 3]. Inflammation is one the most insidious causes of many chronic and overlooked diseases; however, the use of anti-inflammatory drugs (such as steroids and/or nonsteroid drugs) for a long time has in many instances resulted in hormonal side effects and damage to the gastric lesions, kidney, and heart, thereby limiting their uses. Most of these drugs help the immune cells to self-regulate as well as to adjust immune responses to adaptive rather than maladaptive levels [4]. In spite of present-day progress in the development of anti-inflammatory therapy, it is still essential to find potent and effective anti-inflammatories, specifically for the treatment of chronic inflammatory disorders.

In modern medicine, natural products from plants have been used tremendously for the development of important therapeutic drugs [5, 6]. Due to side effects and one target-specific action of allopathic drugs, medicinal herbs or plant natural products work in a way that depends on an orchestral approach. There are multitudes of different molecules in a plant that work coactively on targeted components of the complex cellular pathway [5, 7]. For many centuries, a wide variety of biologically active compounds have been sourced from medicinal plants and utilized magnificently either as pure compounds or as crude material for treating various human and animal disease conditions [6].

*Stemonocoleus micranthus* Harms (Fabaceae) (called Ahianana in Ivory Coast, and Nre and Erhanebeni in Southeast and Edo State, Nigeria, respectively) is an uncommon very large forest tree (up to 45 m high) with leaflets similar to those of *Detarium* species, but with fewer numbers of lateral veins. Its morphology has been previously described [8]. It has a white and hard sapwood and is distributed in the primary forest of southern Nigeria, Congo, eastern Cameroun, Gabon, and Ivory Coast. The stem bark decoction of this plant is most widely used traditionally as remedies for various diseases [9]. Although this plant has not been well investigated, there are reports on its analgesic, narrow-spectrum antibacterial, central nervous system (CNS) depressant, local anesthetic [10], antiulcer [11], antioxidant and hepatoprotective [12], hypolipidemic [13], antimicrobial [14], and antimalarial [15] activities including its use for the treatment of Buruli ulcer lesion in Cameroon [7].

Therefore, the purpose of this research was to evaluate and determine the anti-inflammatory and immunomodulatory activities of *Stemonocoleus micranthus* Harms extract in rats and mice, respectively.

## 2. Materials and Methods

### 2.1. Chemicals, Reagents, Solvents, and Drugs

All chemicals used were of analytical grade and include methanol (Sigma-Aldrich, Germany), dichloromethane (Sigma-Aldrich, Germany), distilled water, chloroform, ketamine hydrochloride (Rotex Medical, Germany), xylazine, xylene, normal saline, 0.1% sodium carbonate solution, Leishman's stain, Indian ink, Hayem solution, levamisole (Ecomed Pharma Ltd, Nigeria), and cyclophosphamide (Kwality Pharma Ltd, India).

### 2.2. Equipment/Instruments

The equipment and instruments used include milling machine (Lab mill, serial no. 4745, Christy and Norris Ltd., England), electronic animal weighing balance (B. Bran Scientific & Instrument Co. England), analytical weighing balance, Soxhlet apparatus, rotary evaporator (B. Bran Scientific & Instruments Co., England), oven, incubator, photoelectronic colorimeter (Mumbai, India), and visible spectrophotometer (Mumbai, India).

### 2.3. Experimental Animals

Adult Swiss albino rats (150–200 g) and Swiss albino mice (17–25 g) of both sexes were obtained from the Animal Facility of the Department of Pharmacology and Toxicology, University of Nigeria, Nsukka. Animal experiments were done in compliance with the National Institute of Health Guide for Care and Use of Laboratory Animals (Pub. no. 85–23, revised 1985).

### 2.4. Collection, Preparation, and Extraction of Plant Material

Fresh *Stemonocoleus micranthus* stem barks were collected from the forest of Orba Nsukka in Udenu Local Government Area of Enugu State, Nigeria, in the month of July. The identification and authentication were done at International Center for Drug Research and Development (InterCEDD), Nsukka, Enugu State, Nigeria, by Mr. Alfred Ozioko. The stem barks were separated from the woody part, sliced into small bits, sun-dried, and pulverized into powder. The bark powder (2.4 kg) was weighed and stored in an airtight container before use. About 2.4 kg of the powdered material was extracted with about 10 L of a 1 : 1 mixture of methanol-dichloromethane by continuous extraction in a Soxhlet extractor. The filtrate was concentrated using a rotary evaporator to obtain methanol-dichloromethane extract of *Stemonocoleus micranthus* (MDESm) (106.28 g). The extract was stored in an amber coloured bottle in a refrigerator at 4°C until its use. Distilled water was the reconstitution solvent for the extract and levamisole.

### 2.5. Phytochemical Analysis of the Extract

The MDESm was subjected to phytochemical analysis using standard procedures [16, 17].

### 2.6. Pharmacological Tests

#### 2.6.1. Acute Toxicity Test

The mean oral lethal dose (LD_50_) of MDESm was estimated in mice using Lorke's method [18].

#### 2.6.2. Carrageenan-Induced Rat Paw Oedema

Twenty-five albino rats were weighed and randomly divided into five groups (*n* = 5). Group I received oral administration of distilled water (2 ml/kg) and group 2 received ibuprofen (100 mg/kg) while groups 3, 4, and 5 received 100, 200, and 400 mg/kg MDESm, respectively. One hour later, 0.1 ml of 1% w/v carrageenan (phlogistic agent) in normal saline was injected into the subplantar region of the right hind paw of the rats, and the volume of the paw size was measured by water displacement at 0 h and at 1, 2, 3, 4, and 5 h after carrageenan injection [19].

The percent inhibition of oedema was calculated using the following formula [20]:

(1) $$\begin{array}{c} \text{inhibition of oedema}\left(\text{\%}\right)=\frac{{\text{V}}_{c}-{\text{V}}_{t}}{{\text{V}}_{c}}\;, \end{array}$$

where *V*_*c*_ is the mean paw oedema volume of control at each hour and *V*_*t*_ is the mean paw volume of treated animals at each hour.

#### 2.6.3. Xylene-Induced Ear Oedema in Mice

Adult albino mice (20–25 g) were randomly allotted into three groups (*n* = 5). Groups 1–3, respectively, received topical administration of 0.05 ml of MDESm (100 mg), ibuprofen (100 mg), and distilled water on the anterior part of the right ear. Thereafter, topical inflammation was instantly induced on the posterior surface of the same ear by application of xylene (50 *μ*l). The left ear was left untreated. Two hours after induction of inflammation, mice were sacrificed by suffocation with chloroform and both ears removed. Circular discs were punched out of the right (treated) and left (untreated) ear lobes using a cork borer (6 mm diameter) and weighed [21]. The difference in the weight of the discs from the right treated and left untreated ears was calculated and used as a measure of oedema relative to control by using the following equation [22]:

(2) $$\begin{array}{c} \text{inhibition of oedema }\left(\text{\%}\right)=\left[1-\left(\frac{R_{t}-L_{t}}{R_{c}-L_{c}}\right)\right]\times 100, \end{array}$$

where *R*_*t*_ is the mean weight of right ear plug of treated animals; *L*_*t*_ is the mean weight of left ear plug of treated animals; *R*_*c*_ is the mean weight of right ear plug of control animals; and *L*_*c*_ is the mean weight of left ear plug of control animals.

#### 2.6.4. Cotton Pellet-Induced Granuloma in Rats

Twenty-five albino rats were weighed and randomly divided into five groups (*n* = 5). The animals were anaesthetized with ketamine (50 mg/kg, p.o.) and xylazine (10 mg/kg, p.o.) before two sterilized cotton pellets (30 mg each) were surgically implanted subcutaneously on either sides in the lumbar region on the dorsal surface of each rat. After the surgery, the animals were allowed to recover before the groups were, respectively, given MDESm (100, 200, and 400 mg/kg), ibuprofen (100 mg/kg), or distilled water (2 ml/kg) orally once daily for seven days. On the 8^th^ day, the animals were sacrificed with chloroform suffocation. The implanted cotton pellets together with the attached granuloma tissues were surgically removed and dried in an oven at 60°C to a constant weight and the weights recorded. The percentage weight increase of granuloma tissue formed was calculated relative to the control [23].

### 2.7. Immunomodulatory Study

#### 2.7.1. Cyclophosphamide-Induced Neutropenia Assay

Swiss albino mice were randomly divided into five groups (*n* = 5), and the groups, respectively, received oral administration of 1 ml/kg distilled water, 50 mg/kg levamisole, and 100, 200, and 400 mg/kg MDESm, for 10 days. Subsequently, a neutropenic dose of cyclophosphamide (200 mg/kg, s.c.) was injected into the animals on the 10^th^ day (the day labelled as day zero). The total leukocyte (TLC) and differential leukocyte counts (DLC) were calculated on day 0 before injection and on day 3 after injection of cyclophosphamide. Blood samples for the determination of TLC and DLC were collected from the animals through the retro-orbital plexus into heparinized tubes. The percentage TLC and DLC in pretreated (day 0) and treated (day 3) groups were compared with the values of the control group [24].

#### 2.7.2. Neutrophil Adhesion Test

Neutrophil adhesion test was performed based on the method described by Fulzele et al. [25]. Twenty-five albino rats were randomly divided into five groups (*n* = 5) and received oral administration of distilled water (1 ml/kg), levamisole (50 mg/kg), and MDESm (100, 200, and 400 mg/kg), respectively, for 10 days. On the 10^th^ day, blood samples were collected from the animals through retro-orbital plexus into heparinized tubes and were analysed for TLC and DLC after slide fixation and staining with Leishman's reagent. After the initial counts, the blood samples were incubated with nylon fibres (80 mg/ml) for 10 min at 37°C. The incubated blood samples were again analysed for TLC and DLC. The percentages of neutrophils in the nylon fibre-treated and nylon fiber-untreated bloods were determined, and the difference was taken as an index of neutrophil adhesion [26, 27]. From the results of DLC, TLC, and neutrophil index, the percent neutrophil adhesion was calculated as follows:

(3) $$\begin{array}{c} \text{neutrophil adhesion }\left(\text{\%}\right)=\frac{{\text{NI}}_{u}-{\text{NI}}_{t}}{{\text{NI}}_{u}}\times 100, \end{array}$$

where NI_*u*_ is the neutrophil index before incubation with nylon fibres and NI_*t*_ is the neutrophil index after incubation with nylon fibres.

#### 2.7.3. Carbon Clearance Test

Phagocytic activity of reticuloendothelial systems (RES) was assayed by the carbon clearance test. In this test, twenty-five albino rats were randomly divided into five groups (*n* = 5); group 1 received oral dose of distilled water (1 ml/kg) and group 2 received levamisole (50 mg/kg) while groups 3, 4, and 5 received MDESm (100, 200, and 400 mg/kg), respectively, for 10 days. After 48 h of the last dose, 0.1 ml Indian ink was injected into the animals via the tail vein and blood was withdrawn from the retro-orbital plexus of the animals at 0 min before and 15 min after injection of the ink. The blood samples (50 *μ*l) were mixed with 4 ml of sodium carbonate solution (0.1%) and the absorbance of the mixture was determined at 660 nm [26, 28]. Phagocytic index, K, which is the rate of carbon elimination from the reticuloendothelial system, was calculated using the following equation:

(4) $$\begin{array}{c} \text{phagocytic index},\text{K}=\;\frac{{\text{log OD}}_{1}-{\text{log OD}}_{2}\;}{15\;}, \end{array}$$

where OD_1_ is the optical density at 0 min and OD_2_ is the optical density at 15 min.

#### 2.7.4. Haematological Parameter Assay

Twenty-five albino mice were randomly divided into five groups (*n* = 5). Group 1 received oral dose of distilled water (1 ml/kg) and group 2 received levamisole (50 mg/kg), while groups 3, 4, and 5 received MDESm (100, 200, and 400 mg/kg), respectively, for 10 days. At the end of the treatment, blood samples were collected from the retro-orbital plexus of the rats using heparinized capillary tubes, and the determination of haematological indices was done using standard procedures [29, 30].

*(1). Total Leucocyte Count (TLC) Test*. This test is also known as the total white blood cell (WBC) count test. The test was carried out by mixing 0.38 ml of 1% glacial acetic acid (the WBC diluting fluid) with 0.02 ml of blood in a test tube, and the resultant mixture was counted with the improved Neubauer counting chamber using ×40 magnification lens. The four corner squares of the central square were counted, and the number of cells (cells/*μ*l) counted was recorded.

*(2). Differential Leucocyte Count (DLC) Test*. The differential leucocyte count test was carried out by fixing the blood on a slide and staining with Leishman's stain. The slides were kept for 8 min before the excess stain was washed off with a sufficient quantity of water and were air-dried. Oil immersion was added on the slides which were subsequently examined under a light microscope using ×100 magnifications. The percentage neutrophils (cells/*μ*l) were determined.

*(3). Red Blood Cell Count Test*. Blood (20 *μ*l) was mixed with 4 ml of Hayem's solution (RBC diluting fluid), and the resultant mixture was counted with the improved Neubauer counting chamber as described for TLC above using ×40 magnification.

*(4). Platelet Determination*. The platelet count test was carried out by mixing 0.38 ml of filtered ammonium oxalate (platelet diluting fluid) with 0.02 ml of blood in a test tube, and the mixture was counted with the improved Neubauer counting chamber using ×40 magnification. The platelets (cells/*μ*l) were counted only on the 16 squares of one square.

*(5). Estimation of the Haemoglobin (Hb) Content*. Cyanmethemoglobin method was adopted. A 4 ml of Drabkin's solution (Hb diluting fluid) was mixed with 0.02 ml of blood in a test tube, allowed to stand for 5 min, and viewed against the blank at a wavelength of 540 nm with a photoelectronic colorimeter. The observed values (g/dl) were read off on a calibration curve.

#### 2.7.5. Assessment of Biochemical Parameters

Twenty-five albino rats were randomly divided into five groups (*n* = 5) and received oral doses of distilled water (1 ml/kg), levamisole (50 mg/kg), and MDESm (100, 200, and 400 mg/kg), respectively, for 10 days. At the end of the treatment, blood samples were collected from the retro-orbital plexus using nonheparinized capillary tubes, and biochemical parameters (liver enzymes) were assayed following the procedures outlined by Kind and King [31, 32].

*(1) Serum Alanine Transaminase (ALT) and Aspartate Transaminase (AST) Determination*. A colorimetric method was used. The two procedures are the same but with their respective substrates. A volume (0.5 ml) of ALT substrate solution (200 mmol/L DL-alanine and 2 mmol/L *α*-ketoglutarate) or AST substrate solution (200 mmol/L L-aspartate (in Tris buffer, pH 7.4) and 2 mmol/L ketoglutarate) was added into five test tubes which were preincubated for 30 min at 37°C. Afterwards, 0.1 ml of serum sample was added to the test tubes which were incubated for 30 min at 37°C. Later, 0.5 ml of the colour developer (1 mmol/L 2, 4-dinitrophenylhydrazine) was added to the sample and the standard solution (1.2 mmol/L pyruvate) test tubes. The tubes were allowed to stand for 20 min at room temperature. Thereafter, 5 ml of 4 N NaOH working solution was added to the test tubes which were then allowed to stand for 15 min at room temperature. The absorbance of both test samples and standard solutions were read at 505 nm against deionized water blank. The experiment was repeated for all the groups. The ALT or AST activity of the samples was obtained by interpolating the absorbance obtained for the samples in the calibration curve made from the standard. The results were expressed in international unit per liter (IU/L) as shown in the following equation:

(5) $$\begin{array}{c} \text{ALT }\left(\text{IU}/\text{L}\right)=\;\frac{{\text{Abs}}_{\text{sample}}-{\text{ Abs}}_{\text{standard}}}{0.059},\\ \text{ AST I}\left(\text{U}/\text{L}\right)=\;\frac{{\text{Abs}}_{\text{sample}}-{\text{ Abs}}_{\text{standard}}}{0.002}. \end{array}$$

*(2). Serum Alkaline Phosphatase (ALP) Determination*. The test was carried out by adding 0.05 ml of serum to 0.5 ml of buffer (pH 10) (premixed with 0.5 ml substrate (M/100 Na_2_-phenyl phosphate) and warmed at 37°C for 3 min) in a tube. Then, 0.8 ml of N/2 NaOH was added followed by 1.2 ml of M/2 NaHCO_3_. Then, 1 ml of 0.6% aminoantipyrine was added and mixed before the addition of 1 ml of 2.4% K_3_Fe(CN)_6_. The resultant mixture was read at a wavelength of 405 nm with a photoelectronic colorimeter. Reading was repeated after 1, 2, and 3 min. Alkaline phosphatase activity (IU/L) was estimated using the following formula:

(6) $$\begin{array}{c} \text{alkaline phosphatase }\left(\text{IU}/\text{L}\right)=\;\frac{{\text{Abs}}_{\text{sample}}-{\text{ Abs}}_{\text{control}}}{{\text{Abs}}_{\text{standard}}-{\text{ Abs}}_{\text{blank}}}\;\times 30. \end{array}$$

### 2.8. Statistical Analysis

The statistical analyses of data obtained were done by one-way analysis of variance (ANOVA) and subjected to Dunnett's post hoc tests. Data were presented as mean ± SEM and significant differences accepted at *p* < 0.05.

## 3. Results

### 3.1. Extractive Yield and Phytochemical Screening

The extraction process yielded 106.28 g (4.4% w/w) of MDESm. The phytochemical analysis of MDESm showed that the plant extract was rich in alkaloids, glycosides, flavonoids, reducing sugars, carbohydrates, steroids, terpenoids, tannins, fats and oil, and proteins (Table 1).

### 3.2. Acute Toxicity Test

The acute toxicity test of MDESm in mice showed no death or sign of acute intoxication after 24 h observation period in both stages of the test. The LD_50_ was estimated to be above 5 g/kg.

### 3.3. Carrageenan-Induced Rat Paw and Xylene-Induced Ear Oedema in Mice

The result of this test showed that MDESm exhibited a non-dose-dependent rat paw oedema reduction with 100 mg/kg causing a significant (*p* < 0.05) oedema reduction from the 3^rd^ hour up till the 5^th^ hour in comparison with control (Table 2). The percentage oedema reduction at this dose is somehow comparable to that of ibuprofen at these hours even though the reduction caused by ibuprofen started from the 2^nd^ hour. From the area under the curve (AUC) values, the global oedema reduction caused by these treatments follows the same pattern as seen in the percentage oedema reduction with MDESm (100 mg/kg) having the lowest area among the other doses of the extract. The order of increasing AUC (mlh) values for the treatments is as follows: 1.270 (ibuprofen) <1.890 (MDESm, 100 mg/kg) <2.820 (MDESm, 200 mg/kg) <3.480 (MDESm, 400 mg/kg) <3.490 (control) (Table 2).

**Figure 1:**
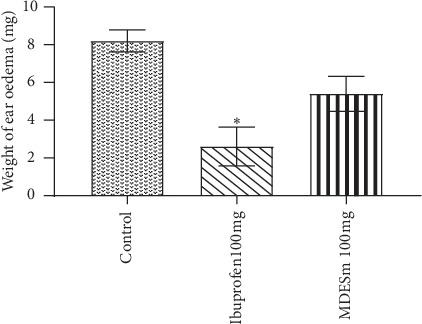
Effect of MDESm on xylene-induced ear oedema in mice (*n* = 5); values were expressed as mean ± SEM. ^*∗*^*p* < 0.05 compared to control (one-way ANOVA; Dunnett's post hoc tests). MDESm: methanol-dichloromethane extract of *Stemonocoleus micranthus*.

Topical application of MDESm (100 mg) reduced xylene-induced ear oedema by 5.4 ± 0.927 (34.1%) although not significantly; ibuprofen (100 mg) significantly (*p* < 0.05) reduced it by 2.60 ± 1.030 (51.0%) when compared to control (8.2 ± 0.583) (Figure 1).

### 3.4. Effect of MDESm on Cotton Pellet-Induced Granuloma

The weight of the granuloma tissue for the control group of animals was found to be 0.28 ± 0.02 mg (Table 3). Treatment with the different concentrations of MDESm reduced the granuloma tissue formed and only MDESm (100 mg/kg) significantly (*p* < 0.05) reduced the weight by 39.28% which was slightly greater than the reduction (32.14%) caused by ibuprofen (Table 3).

### 3.5. Effect of MDESm on Cyclophosphamide-Induced Neutropenia

The administration of cyclophosphamide (200 mg/kg) caused a reduction in neutrophil and total leucocyte counts in most of the groups. The percentage reduction of TLC in the MDESm-treated groups when compared to the control group increased with increasing dose of the extract as follows: 100 mg/kg (68.32%), 200 mg/kg (75.68%), and 400 mg/kg (83.12%), which were lower than the significant (*p* < 0.05) reduction (95.34%) observed in the levamisole-treated group. However, the reduction in neutrophil count did not follow the same pattern as observed in the TLC. The levamisole-treated group recorded the highest percentage reduction of 31.4% followed by MDESm 200 mg/kg with 10.16% while there was no reduction in MDESm 100 mg/kg which had 0%, but there was an increase in MDESm 400 mg/kg group with a negative percentage value of −14.6%, all in comparison to control (Table 4).

### 3.6. Effect of MDESm on Neutrophil Adhesion

Incubation of blood with nylon fibres caused a decrease in the neutrophil counts due to adhesion of neutrophils to the fibres. There was a dose-dependent increase in the percentage of neutrophils that adhered to the nylon fibres in the MDESm-treated groups (100 mg/kg (41.1%), 200 mg/kg (45.04%), and 400 mg/kg (45.78%)); these values were comparable to that of levamisole which had a higher percentage (46.78%) of adhered neutrophils but were significantly (*p* < 0.05) lower than that of control (64.5%) (Table 5).

### 3.7. Effect of MDESm on Carbon Clearance

The carbon clearance method was used to study the *in vivo* phagocytic activity of MDESm. The MDESm 100 and 200 mg/kg caused higher phagocytosis with phagocytic indices of 0.0447 ± 0.00762 and 0.0466 ± 0.00703, respectively, more than that of MDESm 400 mg/kg with a phagocytic index of 0.0096 ± 0.00903 and levamisole (0.0333 ± 0.01794). These values were not significant when compared to the value (0.0226 ± 0.02117) for control (Table 6).

### 3.8. Effect of MDESm on Haematological Parameters

Administration of MDESm (100, 200, and 400 mg/kg) in rats for 10 days did not have any significant effect on the haematological indices of the animals in comparison with the control group (Table 7).

### 3.9. Effect of MDESm on Biochemical Parameters in Rats

From Table 8, there were a dose-dependent decrease in ALT of rats treated with MDESm (100, 200, and 400 mg/kg) and a significant (*p* < 0.05) increase in AST caused by 400 mg/kg which was greater than that of levamisole (55.4 ± 9.21) and other doses of the extract (100 mg/kg (49.3 ± 13.33) and 200 mg/kg (45.6 ± 3.04)) in comparison with control (48.2 ± 5.60). However, MDESm at all doses caused a significant (*p* < 0.05) reduction in ALP compared to control.

## 4. Discussion

In this study, the anti-inflammatory and immunomodulatory activities of *Stemonocoleus micranthus* Harms (Fabaceae) stem bark extract were investigated using different experimental models.

Preliminary screening for the presence of secondary metabolites in plant extract is an important step for quality control and standardization. This study has shown that MDESm is rich in active metabolites such as alkaloids, saponins, flavonoids, steroids, tannins, and terpenoids as previously reported [11, 12], which may be responsible for its mild anti-inflammatory and immunomodulatory activities. It has been reported that flavonoids possess many pharmacological properties such as immunomodulatory, anti-inflammatory, and antioxidant activities [33–36]. The acute toxicity test showed that MDESm was safe above the dose of 5000 mg/kg, indicating high degree of safety from acute intoxication.

The *in vivo* anti-inflammatory activity was investigated using carrageenan-induced rat paw oedema. Carrageenan-induced rat paw oedema is largely associated with the production of several inflammatory mediators such as histamine, prostaglandins, kinins, nitric oxide (NO), and cytokines [37]. The development of carrageenan-induced paw oedema is biphasic in nature [38]. The first phase (1-2 h after carrageenan injection) is mediated by histamine, serotonin, and bradykinins released from mast cells while the second phase (3–5 h after carrageenan injection) is connected with the release of arachidonate metabolites such as leukotrienes and cytokines [39–41]. The groups pretreated with MDESm (100 mg/kg, p.o.) significantly reduced rat paw oedema from the 3^rd^ to the 5^th^ h post-carrageenan injection, compared with the control group showing that its anti-inflammatory activity may be at much lower doses.

The acute anti-inflammatory activity of any agent can be estimated by xylene-induced ear oedema test [22]. Xylene is a phlogistic agent that increases oedema formation by initiating the actions of mediators such as serotonin, histamine, bradykinin, and prostaglandins causing inflammation [42]. Findings from this test indicate that MDESm at a dose of 100 mg had mild anti-inflammatory activity as it reduced the xylene-induced ear oedema in the treated mice although not significantly.

Cotton pellet induced-granuloma model is used to assess the effect of extract in chronic inflammation [43]. It is used to evaluate the transudative and proliferative components of chronic inflammation [44, 45]. Several herbs have been reported to interfere with the synthetic pathway of proinflammatory mediators responsible for chronic inflammation by inhibition of lipoxygenase which is an important enzyme in arachidonic acid pathway [46]. However, the weight of granuloma tissue is determined by the amount of granuloma deposited on the cotton pellets together with the fluid absorbed. The weight of wet cotton pellets correlates with exudative material and weight of the dry pellets correlates with the amount of granuloma tissue deposited [47, 48]. MDESm at a dose of 100 mg/kg showed a significant (*p* < 0.05) decrease in the granuloma tissue deposited on the cotton compared to control, with percentage reduction of granuloma tissue slightly greater than that of ibuprofen. With increasing doses of MDESm, there was a gradual increase in the weight of deposited granuloma tissue, indicating that it is more effective at a lower dose. In other words, MDESm inhibited chronic inflammation in a non-dose-dependent manner.

In experimental animals, cyclophosphamide is usually used to induce myeloid cell suppression. As an alkylating agent, it functions as an immunosuppressive agent by causing alkylation of DNA, in turn by interfering in DNA synthesis and function [49]. MDESm at a high dose (400 mg/kg, p.o.) abolished the cyclophosphamide-induced neutropenia, suggesting that it may be affecting the hemopoetic system by causing immunostimulation. The activation of macrophages, which release a number of mediators including colony-stimulating factor and interleukin, could be the way cyclophosphamide-induced neutropenia may be prevented [50]. Neutrophil and monocytes are key players in innate immune response, and the results of this study show that MDESm strengthens the immune system.

The adhesion of neutrophil to nylon fibres indicates the migration of the immune cells in the blood vessels and the number of neutrophils reaching the site of inflammation [50]. MDESm and levamisole drug did not increase significantly the amount of neutrophils adhered to the nylon fibre compared to the control group. Hence, it can be inferred that MDESm did not cause appreciable stimulation of neutrophils towards the site of inflammation which may suggest weak immunostimulatory potential, although its immunostimulation increases with increasing dose. Determination of the performance of the reticuloendothelial system and its granulopoetic activity is based on the rate of carbon clearance [51]. Thus, the quicker removal of carbon particles from the blood is related to the rise in phagocytic activity. The MDESm at lower doses enhanced the phagocytic function by clearing the carbon particle more than levamisole which is a standard immunostimulatory agent. However, this suggests its potential to stimulate the reticuloendothelial system.

The hematopoietic stem cells in the bone marrow are the progenitor of most of the immune system cells. It is in this microenvironment that the site of antigen-dependent differentiation of B-lymphocytes is located [50]. Treatment of mice with MDESm for 10 days did not have any significant increase or decrease in their haematological parameters compared to the control group, although at intermediate dose, there was a slight reduction in WBC which may suggest mild immunosuppression at that dose.

Some of the most sensitive markers of hepatocellular injury are ALT and AST, and their elevation in serum is indicative of cellular leakage and loss of the functional integrity of cell membranes in the liver [52]. In this study, the increase in AST which may be from other sources apart from liver and a dose-dependent decrease in ALT after MDESm administration indicated mild deterioration and nondeleterious effect, respectively, on the cellular integrity and status of hepatic cells. ALP is a membrane-bound enzyme involved in active transport across the capillary wall [52]. The observed decrease in ALP activity in the MDESm-treated groups in comparison with the control group is also an indication of hepatoprotection. Overall, the positive effect of MDESm on biochemical parameters is in tandem with the findings of the previous study on the hepatoprotective potentials of this extract against CCl_4_-induced oxidative liver damage [12].

## 5. Conclusion

The observed non-dose-dependent and mild anti-inflammatory and immunomodulatory activities (which may increase with fractionation) of the methanol-dichloromethane stem bark extract of *Stemonocoleus micranthus* Harms seem to support the use of this plant in the treatment of various diseases and metabolic disorders. These activities may largely be as a result of single or combined effects of bioactive compounds present in the plant. However, further investigation is required to isolate the phytoconstituents responsible for the observed activities and possibly elucidate its mechanism of anti-inflammatory and immunomodulatory potential.

## Figures and Tables

**Table 1:** List of compounds present in MDESm.

Constituents	Relative presence
Alkaloids	+
Carbohydrates	++++
Fats and oils	++
Flavonoids	++++
Glycosides	++++
Proteins	+++
Reducing sugars	+++
Resins	—
Saponins	++
Steroids	++
Tannins	++++
Terpenoids	+

— = not present; + = present in small concentration; ++ = present in moderate concentration; +++ = present in high concentration; ++++ = abundantly present; MDESm = methanol-dichloromethane extract of *Stemonocoleus micranthus*.

**Table 2:** Effect of MDESm on carrageenan-induced rat paw oedema.

Treatment	Dose (mg/kg)	1 h	2 h	3 h	4 h	5 h	AUC (mlh)
		Oedema (ml)					
Control (dist. water)	2 ml/kg	0.42 ± 0.10	0.76 ± 0.074	0.82 ± 0.047	1.00 ± 0.063	0.98 ± 0.058	3.490 ± 0.245
Ibuprofen	100	0.16 ± 0.07 (61.9)	0.28 ± 0.09^*∗*^ (63.2)	0.30 ± 0.12^*∗*^ (63.4)	0.36 ± 0.12^*∗*^ (64.0)	0.33 ± 0.11^*∗*^ (66.32)	1.270 ± 0.345
MDESm	100	0.24 ± 0.02 (42.9)	0.52 ± 0.09 (31.6)	0.40 ± 0.14^*∗*^ (51.2)	0.52 ± 0.15^*∗*^ (48.0)	0.42 ± 0.14^*∗*^ (57.1)	1.890 ± 0.392
MDESm	200	0.30 ± 0.09 (28.6)	0.66 ± 0.11 (13.2)	0.78 ± 0.14 (4.9)	0.70 ± 0.13 (30.0)	0.76 ± 0.12 (22.5)	2.820 ± 0.396
MDESm	400	0.34 ± 0.07 (19.1)	0.75 ± 0.15 (1.3)	0.94 ± 0.06 (−14.6)	0.98 ± 0.04 (2.0)	0.92 ± 0.05 (6.1)	3.480 ± 0.289

Mean ± SEM, *n* = 5; *p* < 0.05 compared to control (one-way ANOVA; Dunnett's post hoc test); values in parentheses are percentage oedema reduction compared to control. MDESm = methanol-dichloromethane extract of *Stemonocoleus micranthus*; AUC = area under the curve.

**Table 3:** Effect of MDESm on cotton pellet-induced granuloma in rats.

Treatment	Dose (mg/kg)	Mean weight (mg)	% reduction
Control (dist. water)	2 (ml/kg)	0.28 ± 0.02	—
Ibuprofen	100	0.19 ± 0.032^*∗*^	32.14
MDESm	100	0.17 ± 0.016^*∗*^	39.28
MDESm	200	0.23 ± 0.029	17.85
MDESm	400	0.23 ± 0.012	17.85

Mean ± SEM, *n* = 5; ^*∗*^*p* < 0.05 compared to control (one-way ANOVA; Dunnett's post hoc tests). MDESm = methanol-dichloromethane extract of *Stemonocoleus micranthus*.

**Table 4:** Effect of MDESm on cyclophosphamide-induced neutropenia in mice.

Treatment	Dose (mg/kg)	Total leucocyte count (cells/mm^3^)		Reduction in cell number after cyclophosphamide treatment (%)	Total neutrophils (cells/mm^3^)		Reduction in cell number after cyclophosphamide treatment (%)
		Before	After		Before	After	
Control (dist. water)	1 (ml/kg)	9120 ± 1563	1180 ± 162	7940 ± 1573 (87.06)	22.4 ± 3.74	24.6 ± 2.80	−2.2 ± 1.88 (−9.82)
Levamisole	50	15880 ± 3172	740 ± 163	15140 ± 3315^*∗*^ (95.34)	21.0 ± 3.79	14.4 ± 2.15	6.6 ± 1.80 (31.40)
MDESm	100	10575 ± 1003	3350 ± 212	7225 ± 3078 (68.32)	18.7 ± 3.42	18.7 ± 3.06	0.0 ± 5.11 (0.00)
MDESm	200	7980 ± 1285	1940 ± 821	6040 ± 1557 (75.68)	23.6 ± 3.50	21.2 ± 3.12	2.2 ± 4.03 (10.16)
MDESm	400	9925 ± 726	1675 ± 342	8250 ± 1034 (83.12)	22.2 ± 3.81	25.5 ± 2.90	−3.3 ± 5.55 (−14.86)

Mean ± SEM; *n* = 5, ^*∗*^*p* < 0.05 compared to control (one-way ANOVA; Dunnett's post hoc test). MDESm = methanol-dichloromethane extract of *Stemonocoleus micranthus*.

**Table 5:** Effect of MDESm on neutrophil index and neutrophil adhesion in rats.

Treatment	Dose (mg/kg)	TLC (cells/mm^3^) (*x*)		% neutrophil (*y*)		Neutrophil adhesion (%)		Neutrophil index (*xy*)
		UnB	FTB	UnB	FTB	UnB	FTB	
Control (dist. water)	1 (ml/kg)	11128 ± 1593	6180 ± 1194	24.2 ± 3.32	15.0 ± 2.19	272000 ± 51722	96560 ± 25850	64.50
Levamisole	50	8040 ± 1073	6500 ± 1121	19.6 ± 1.91	12.4 ± 1.12	156280 ± 20293	83160 ± 20426	46.78
MDESm	100	13900 ± 1100	11475 ± 891^*∗*^	28.8 ± 1.49	20.5 ± 70	401000 ± 43287^*∗*^	236050 ± 27009	41.13^*∗*^
MDESm	200	15440 ± 3068	11140 ± 1573^*∗*^	24.0 ± 3.98	18.2 ± 3.69	346400 ± 54386	190380 ± 34520	45.04^*∗*^
MDESm	400	9560 ± 927	7700 ± 559	23.0 ± 3.82	15.0 ± 3.17	210880 ± 17759	114280 ± 25910	45.80

Mean ± SEM, *n* = 5; ^*∗*^*p* < 0.05 compared to control (one-way ANOVA; Dunnett's post hoc test). UnB: untreated blood; FTB: nylon fibre-treated blood; MDESm: methanol-dichloromethane extract of *Stemonocoleus micranthus*.

**Table 6:** Effect of MDESm on phagocytic index in carbon clearance assay in rats.

Treatment	Dose (mg/kg)	Phagocytic index
Control (dist. water)	—	0.0226 ± 0.02117
Levamisole	50	0.0333 ± 0.01794
MDESm	100	0.0447 ± 0.00762
MDESm	200	0.0466 ± 0.00703
MDESm	400	0.0096 ± 0.00903

Mean ± SEM, *n* = 5; ^*∗*^*p* < 0.05 compared to control (one-way ANOVA; Dunnett's post hoc tests). MDESm = methanol-dichloromethane extract of *Stemonocoleus micranthus*.

**Table 7:** Effect of MDESm on haematological parameters in mice.

Treatment	Dose (mg/kg)	WBC (cells/*μ*L)	Hb (g/dL)	Platelets (cells/*μ*L)	Neutrophil (cells/*μ*L)	Lymphocytes (cells/*μ*L)
Control (dist. water)	—	9120 ± 1563	13.38 ± 0.31	222000 ± 48104	22.4 ± 3.74	77.6 ± 3.74
Levamisole	50	15880 ± 3172^*∗*^	13.46 ± 0.44	388000 ± 77549	21.0 ± 3.79	79.0 ± 3.79
MDESm	100	10575 ± 1003	11.90 ± 0.40	255000 ± 71821	18.7 ± 3.42	81.3 ± 3.42
MDESm	200	7980 ± 1285	13.90 ± 0.85	200000 ± 35777	23.6 ± 3.50	76.8 ± 3.73
MDESm	400	9925 ± 726	13.49 ± 0.68	217500 ± 51700	22.2 ± 3.81	77.5 ± 3.81

Mean ± SEM, *n* = 5; ^*∗*^*p* < 0.05 compared to control (one-way ANOVA; Dunnett's post hoc test). MDESm = methanol-dichloromethane extract of *Stemonocoleus micranthus*.

**Table 8:** Effect of MDESm on biochemical parameters in rats.

Treatment	Dose (mg/kg)	AST (IU/L)	ALT (IU/L)	ALP (IU/L)
Control (dist. water)	−50	48.2 ± 5.60	22.7 ± 3.22	40.7 ± 7.98
Levamisole		55.4 ± 9.21	24.2 ± 3.18	46.2 ± 8.64
MDESm	100	49.3 ± 13.33	19.3 ± 2.02^*∗*^	21.6 ± 13.90^*∗*^
MDESm	200	45.6 ± 3.04	15.8 ± 2.57^*∗*^	15.8 ± 9.89^*∗*^
MDESm	400	59.0 ± 11.32^*∗*^	13.0 ± 5.70	37.6 ± 10.00^*∗*^

Mean = ± SEM, *n* = 5; *p* < 0.05 compared to control (one-way ANOVA; Dunnett's post hoc test). MDESm = methanol-dichloromethane extract of *Stemonocoleus micranthus*; ALT = alanine transaminase; ALP = alkaline phosphatase; AST = aspartate transaminase.

## Data Availability

The experimental data obtained from various rat models used to support the findings of this study are available from the corresponding author upon request.
